# Development of a Diet Quality Index Adapted for Pregnant Women

**DOI:** 10.11606/S1518-8787.2018052000184

**Published:** 2018-05-07

**Authors:** Lívia Castro Crivellenti, Daniela Cristina Candelas Zuccolotto, Daniela Saes Sartorelli

**Affiliations:** I Universidade de São Paulo. Faculdade de Medicina de Ribeirão Preto. Programa de Pós-Graduação em Saúde na Comunidade. Ribeirão Preto, SP, Brasil; II Universidade de São Paulo. Faculdade de Medicina de Ribeirão Preto. Departamento de Medicina Social. Ribeirão Preto, SP, Brasil

**Keywords:** Pregnant Women, Prenatal Nutrition, Food Consumption, Healthy Diet, classification, Diet, Food, and Nutrition, Gestantes, Nutrição Pré-Natal, Consumo de Alimentos, Dieta Saudável, classificação, Alimentos, Dieta e Nutrição

## Abstract

**OBJECTIVE:**

To develop a Diet Quality Index Adapted for Pregnant Women (IQDAG) and to evaluate its relation with the characteristics of women treated at the Brazilian Unified Health System.

**METHODS:**

The data on food intake come from a cross-sectional study carried out with 785 adult pregnant women in the city of Ribeirão Preto, state of São Paulo, Brazil, between 2011 and 2012. The index was based on the recommendations of the Brazilian Ministry of Health, previous national dietary indexes, and the new Dietary Guidelines for the Brazilian Population. We used the ANOVA, Kruskal-Wallis, and chi-square tests to describe the quality of the diet according to the characteristics of the mother.

**RESULTS:**

The IQDAG has nine components, and it is represented by three food groups (in servings/1,000 kcal), five nutrients, and a moderator component. A high proportion of pregnant women reached the maximum score for the components of legumes and vegetables. However, few women reached the maximum score for consumption of fresh fruits, fiber, omega-3, calcium, folate, iron, and ultra-processed foods. We verified a better quality of diet among older and eutrophic pregnant women who reported practicing more physical activity and taking dietary supplements. We also observed the highest index score among women with higher intake of carbohydrates, proteins, vitamins C, E, and A, and minerals calcium, folate, and iron, as well as among those with lower intake of total fats and saturated fats.

**CONCLUSIONS:**

This dietary index is unprecedented in incorporating the recommendation of the new Dietary Guidelines for the Brazilian Population regarding the moderation of the consumption of ultra-processed foods. It was useful in evaluating the quality of the diet of pregnant women and we verified a higher score among older and eutrophic women who reported a healthy lifestyle. Strategies are needed to promote a higher consumption of fresh fruits, foods high in fiber, omega-3, calcium, folate, iron, and minimally processed foods in pregnant women.

## RESUMO

**OBJETIVO:**

Desenvolver um Índice de Qualidade da Dieta Adaptado para Gestantes (IQDAG) e avaliar sua relação com características de mulheres atendidas pelo Sistema Único de Saúde.

**MÉTODOS:**

Os dados de consumo alimentar são provenientes de um estudo transversal conduzido entre 785 gestantes adultas do município de Ribeirão Preto, SP, entre 2011 e 2012. A elaboração do índice foi baseada nas recomendações do Ministério da Saúde, em índices dietéticos nacionais prévios e no novo Guia Alimentar para a População Brasileira. Para descrever a qualidade da dieta segundo as características maternas, foram empregados os testes ANOVA, Kruskal-Wallis e qui-quadrado.

**RESULTADOS:**

O IQDAG apresenta nove componentes, sendo representados por três grupos de alimentos (em porções/1.000 kcal); cinco nutrientes; e um componente moderador. Elevada proporção de gestantes atingiu a pontuação máxima para os componentes leguminosas e hortaliças. Porém, poucas mulheres atingiram a pontuação máxima para o consumo de frutas frescas, ingestão de fibras, ômega 3, cálcio, folato, ferro e alimentos ultraprocessados. Verificou-se melhor qualidade da dieta entre gestantes de maior idade, eutróficas, que relataram praticar mais tempo de atividade física e que faziam uso de suplementos dietéticos. A maior pontuação do índice também foi observada entre as mulheres com maior ingestão de carboidratos, proteínas, vitaminas C, E e A, e dos minerais cálcio, folato e ferro, bem como entre aquelas com menor ingestão de gorduras totais e gorduras saturadas.

**CONCLUSÕES:**

O presente índice dietético foi inédito ao incorporar a recomendação do novo Guia Alimentar para a População Brasileira quanto à moderação do consumo de alimentos ultraprocessados. Demonstrou-se útil na avaliação da qualidade da dieta de gestantes e uma maior pontuação foi verificada entre mulheres de maior idade, eutróficas e que relataram um estilo de vida saudável. Estratégias de promoção do consumo de frutas frescas, alimentos ricos em fibras, ômega 3, cálcio, folato, ferro e minimamente processados em gestantes são necessárias.

## INTRODUCTION

Diet indexes are based on theoretical assumptions about the effect of various components of the diet (foods and nutrients) on the health of individuals, which allow the evaluation and monitoring of the adherence to the diet in relation to nutritional recommendations^17^. Their use requires specific adaptations according to the study population^4^. Thus, several international indexes have been developed or adapted for pregnant women, considering the different nutritional needs in this life cycle and the cultural differences of diets between countries^4^ ^,^ ^12^ ^,^ ^19^.

In Brazil, we identified only two studies that adapted diet indexes for pregnant women: the *Índice de Qualidade da Dieta para Gestantes* (IQD-G)^a^ and the *Índice de Alimentação Saudável para Gestantes Brasileiras* (HEIP-B)^20^. The IQD-G was an adaptation of a national index developed to evaluate the quality of diet of all age groups, with the exclusion of children under one year, proposed by Fisberg et al.^7^ The HEIP-B was developed based on the Alternative Healthy Eating Index for Pregnancy (AHEI-P)^26^, with some aspects adapted to the reality of Brazilian pregnant women^20^. However, these indexes have not considered the energy density in the calculation of food groups, which has long been recommended in the scientific literature^10^.

In addition, it is important to incorporate the updates of dietary guidelines into the analysis of the quality of the diet of individuals. The new Food Guide for the Brazilian Population published in 2014^b^ highlights the restriction of the consumption of ultra-processed foods among its recommendations, from the evidence of its association with obesity and chronic non-communicable diseases^21^ ^,^ ^23^.

Evidence suggests that a diet high in vegetables, legumes, and fruits has a better quality and, consequently, promotes positive outcomes for the health of the mother and the baby^12^ ^,^ ^18^ ^,^ ^19^ ^,^ ^28^. Associated with these food groups and micronutrients of interest during the gestational period (iron, calcium, and folate), the adequate intake of omega-3 to favor the maternal and child health is also currently emphasized^6^. Studies suggest that the higher intake of this nutrient in pregnancy is inversely associated with the risk of detrimental outcomes for the mother and baby^3^ ^,^ ^6^.

Given the gaps in the previous Brazilian indexes for pregnant women and the importance of considering the updates of the current dietary guidelines and micronutrients of interest in the gestational period, the objective of this study was to develop a Diet Quality Index Adapted for Pregnant Women (IQDAG) and to evaluate its relation with the socio-demographic characteristics, the lifestyle, and the presence of morbidities of the women treated at the Brazilian Unified Health System (SUS). In addition, we also investigated the correlation of the IQDAG with nutrients of interest and the scores of each component of the index.

## METHODS

In this study, we used data from a cross-sectional study carried out with 785 adult pregnant women in the 2011 and 2012, whose objective was to investigate the relation between the estimation of nutrients of the diet of women users of the SUS in the city of Ribeirão Preto, state of São Paulo, Brazil, and gestational diabetes. A detailed description of the cross-sectional study can be found in the publication of Barbieiri et al.^3^

Information on age, education, self-reported race, and marital status of the pregnant woman were obtained from a structured questionnaire. For the economic classification, we used the Brazilian Economic Classification Criterion (CCEB), which is based on the possession of items and the degree of instruction of the head of the family. The categorization of social classes varies from A (highest level) to E (lowest level)^c^. We also asked the pregnant women about the use of dietary supplements, smoking, physical exercise, and walking in minutes per week. The weight (kg) was measured in a portable digital scale (Tanita, model HS 302) and height (m) was measured in a portable stadiometer (Sanny, model ES 2014). Body mass index (BMI), according to gestational week, was classified using the criteria proposed by Atalah et al.^1^ Gestational age was calculated based on the date of the last menstrual period (LMP) on the pregnancy record.

The diagnosis of gestational diabetes (GD) was performed according to the criteria of the World Health Organization of 2014^30^. We obtained a blood sample after 12 hours of fasting, followed by the ingestion of an overload of 75 g of glucose by the pregnant woman. We determined fasting glycemia one and two hours after the overload using the glucose oxidase test. The presence of hypertension during pregnancy was investigated from the self-report of the pregnant woman.

We estimated food intake, obtained between the 24th and 39th weeks of the pregnancy, using two 24-hour recalls (24hR) surveys on non-consecutive days and a food frequency questionnaire (FFQ)^22^, previously developed and validated for pregnant women^2^.

The 24hR were obtained following the multiple-pass^16^ method, with at least one week between replications.

The FFQ, with 85 food items, was previously developed for pregnant women users of basic health units (BHU)^22^ of the city of Ribeirão Preto, state of São Paulo, Brazil, and its validity was accurate to estimate the dietary intake of the evaluated women^2^.

We used the Brazilian Chemical Composition Table (TACO)^d^ to estimate the nutrients investigated, with the exception of folate, for which we use the table of the United States Department of Agriculture Research Service (USDA)^e^. To calculate the nutritional value of the food, we used the NutWin^®^ Program^f^. We calculated the basal metabolic rate (BMR) of the women using predictive equations and we adopted the Goldberg method^8^ to estimate the sub-report of energy intake (EI). The result of the EI:BMR ratio ≤ 1.35 was considered as a sub-report.

We used the Multiple Source Method (MSM) to estimate the usual diet. The MSM is a program of statistical modeling technique, developed by the European Prospective Investigation into Cancer and Nutrition (EPIC)^13^. This method estimates the usual intake of food and nutrients in three steps. First, it estimates the probability of the intake of some food or nutrient on a random day; second, it estimates the usual intake on the days of consumption; third, the product of the probability of the intake on a random day (first stage) by the usual intake on a day of consumption (second stage) results in the usual intake of individuals^13^ ^,^ ^14^. In addition, the MSM allows us to combine data from short-term dietary surveys, such as the 24hR, with the frequency of data on food consumption from the FFQ^13^. In this study, we obtained the data on food consumption of all pregnant women using the 24hR, corrected for the frequency of consumption reported in the FFQ; on the other hand, we considered all pregnant women as consumers for the estimation of nutrients.

To evaluate the quality of the diet of the pregnant women, we developed the IQDAG based on the recommendations of the Ministry of Health^g^ (2012), as well as the *Índice de Qualidade da Dieta Revisado* (IQD-R)^24^ for the Brazilian population, the *Índice de Alimentação Saudável para Gestantes Brasileiras* (HEIP-B)^20^, and the Dietary Guidelines for the Brazilian Population^b^.

We used the guidelines of the ten steps of healthy eating for pregnant women recommended by the Ministry of Health^g^ to establish the number of servings of the food groups of “Vegetables”, “Legumes”, and “Fresh fruits” (which includes only fruits). According to recommendations of the Health Ministry^g^, the number of servings of food groups is based on a 2,000 kcal diet. We highlight that, in this instrument, we defined the number of recommended daily servings of food groups as 1,000 kcal, similar to the IQD-R^24^. Thus, for every 1,000 kcal of the diet, we adopted the consumption of 1.5 servings of “Vegetables”, 0.5 serving of “Legumes”, and 1.5 servings of “Fresh Fruits”.

We used the HEIP-B^20^ as a reference to define the nutrients of interest - calcium, folate, iron, and fiber -, but with different cut-off points^15^. In addition, considering the evidence on the beneficial effects of omega-3 on maternal and fetal health^6^, we incorporated it as a component of the IQDAG. We highlight that the micronutrient estimate was based on both the diet and use of dietary supplements.

Similar to the Healthy Eating Index 2010 (HEI-2010)^11^ and the IQD-R^24^, this study proposes a moderator component for the index. Following the recommendations of the new Dietary Guidelines for the Brazilian Population^b^, we adopted as moderator component the percentage of total energy value (TEV) from ultra-processed foods. These products refer to industrial formulations made from substances derived from food or synthesized from other organic sources. Generally, these foods contain little or no whole ingredient, are ready to be consumed or heated, and are high in fats, salt, and sugars. Examples include: sugary drinks, cookies, breads with additives, instant noodles, ice cream, chocolates, and frozen and ready-to-heat products^21^. In Brazil, there is no specific recommendation for the consumption of ultra-processed foods. Thus, the cut-off points were based on the 16th and 85th percentile of the distribution curve of the intake of ultra-processed foods by the study population, which correspond to the consumption of 18% and 45% of the TEV of these products, respectively. These cut-off points were established following the proposal of HEI-2010^11^.

We calculated the scores for each component based on the equations described by Melere et al.^20^ In this index, the first equation was used to determine all components of adequacy, which are represented by the food groups of “Vegetables”, “Legumes”, and “Fresh fruits” (servings/1,000 kcal) and the nutrients of “Fibers”, “Omega-3”, “Calcium”, “Folate”, and “Iron”. For an intake greater than or equal to the cut-off points established for these food groups and nutrients, we assigned a maximum score of 10 points and zero for no consumption. The second equation was used to define the moderator component, which represents the percentage of total calories from ultra-processed foods. In this case, the higher the consumption of this component, the lower its score, with zero being the minimum score and 20 points the maximum value. We calculated the intermediate values of the components proportionally and, the final score of the index, which is the sum of all components, has a maximum value of 100 points.

$$\text{Equation}1=\frac{10*(\text{ACCx}-\text{Min})}{\left(\text{Max}-\text{Min}\right)}$$

$$\text{Equation}2=\frac{20*(\text{Min}–\text{ACCx})}{\left(\text{Min}–\text{Max}\right)}$$

In the two equations, ACCx corresponds to the amount consumed of the component x, Min represents the criterion for the minimum score, and Max is the criterion for the maximum score^20^.

To describe the IQDAG score (in tertiles) according to the characteristics of the pregnant women and the dietary estimate, we used the ANOVA (continuous variables with normal distribution), Kruskal-Wallis (continuous variables without normal distribution), and chi-square tests (categorical variables).

We evaluated the correlation between the scores of the components of the IQDAG and the final score using Spearman's correlation coefficient, which we also used to investigate the correlation between the estimation of the diet energy and nutrients and the final score of the index.

We analyzed the data using the SPSS software (SPSS Software, version 17.0) and we adopted the level of significance of p < 0.05.

This study was approved by the Research Ethics Committee of the Centro de Saúde Escola of the Faculdade de Medicina de Ribeirão Preto, of Universidade de São Paulo (Process CEP/CSE-FMRP-USP – 034/2014). All the pregnant women who agreed to participate in the study signed the informed consent.

## RESULTS

The IQDAG has nine components: three food groups (servings/1,000 kcal), (“Vegetables”, “Legumes”, and “Fresh fruits”), five nutrients (“Fibers”, “Omega-3”, “Calcium”, “Folate”, and “Iron”), and one moderator component (percentage of total energy value from ultra-processed foods), as presented in Table 1.

**Table 1:** Components and criteria for the score of the Diet Quality Index Adapted for Pregnant Women (IQDAG)^a^. Ribeirão Preto, state of São Paulo, Brazil, 2012. (n = 785)

Component	Score		
	0	10	20
Vegetables/1,000 kcal (in servings)	0	≥ 1.5	
Legumes/1,000 kcal (in servings)	0	≥ 0.5	
Fresh fruits/1,000 kcal (in servings)	0	≥ 1.5	
Fibers (g)	0	≥ 28	
Omega-3^b^ (mg)	0	≥ 1.4	
Calcium^b^ (mg)	0	≥ 800	
Folate^b^ (μg)	0	≥ 520	
Iron^b^ (mg)	0	≥ 22	
Ultra-processed foods (% TEV)	≥ 45		≤ 18

TEV: total energy value

a Proposed index to evaluate the quality of the diet of pregnant women.

b Estimates from the diet and use of dietary supplements.

The average score (standard deviation) of the index was 70.2 (11.9), ranging from 31.9 to 98.6 points. Regarding the components, we observed that 67.9% of the pregnant women reached the maximum score for the consumption of vegetables, 90.3% for legumes, 18.3% for fresh fruits, 24.7% for fiber, 11.6% for omega-3, 13.6% for calcium, 21.1% for folate, 59.9% for iron, and 15.3% for ultra-processed foods.

Table 2 presents the median (P25; P75) of the food groups and nutrients of the IQDAG.

**Table 2:** Median (P25; P75) of the intake of food groups and nutrients of the Diet Quality Index Adapted for Pregnant Women (IQDAG). Ribeirão Preto, state of São Paulo, Brazil, 2012. (n = 785)

Component	Median (P25; P75)
Vegetables (g)	79.5 (45.3; 110.7)
Legumes (g)	96.1 (67.4; 149.3)
Fresh fruits (g)	92.6 (38.4; 171.8)
Fibers (g)	22.3 (17.5; 28.0)
Omega-3 (mg)	1.0 (0.78; 1.2)
Calcium (mg)	508.2 (368.1; 673.4)
Folate (μg)	393.5 (323.9; 503.1)
Iron (mg)	65.0 (8.8; 69.5)
Ultra-processed foods (% TEV)	31.7 (22.4; 41.3)

TEV: total energy value

We observed that the final score of the IQDAG showed a significant correlation with the score of all components of the index, ranging from 0.23 for the group of legumes to 0.67 for the energy percentage from ultra-processed foods (Table 3).

**Table 3:** Median (P25; P75) of the score of the components of the Diet Quality Index Adapted for Pregnant Women (IQDAG) and its correlation with the final score. Ribeirão Preto, state of São Paulo, Brazil, 2012. (n = 785)

Component	Median (P25; P75)	(r)*	p
Vegetables	10.0 (8.3; 10.0)	0.39	< 0.001
Legumes	10.0 (10.0; 10.0)	0.23	< 0.001
Fresh fruits	5.1 (2.1; 8.9)	0.51	< 0.001
Fibers	8.0 (6.3; 10.0)	0.50	< 0.001
Omega-3	6.9 (5.6; 8.6)	0.25	< 0.001
Calcium	6.4 (4.6; 8.4)	0.34	< 0.001
Folate	7.6 (6.2; 9.7)	0.34	< 0.001
Iron	10.0 (4.0; 10.0)	0.39	< 0.001
Ultra-processed foods	9.8 (2.7; 16.8)	0.67	< 0.001

* Spearman's correlation coefficient between the scores of each component of the IQDAG and the final score.

We identified that women in the highest tertile of the score of the IQDAG had a higher average age, practiced more physical activities, used dietary supplements, and had normal weight, according to the gestational week. We observed no difference in both index score and self-reported hypertension during pregnancy, as well as diagnosis of GD (Table 4).

**Table 4:** Characteristics of the pregnant women according to the score of the Diet Quality Index Adapted for Pregnant Women (IQDAG). Ribeirão Preto, state of São Paulo, Brazil, 2012. (n = 785)

Variable		IQDAG			p^a^
		1st Tertile (n = 261)	2nd Tertile (n = 263)	3rd Tertile (n = 261)	
Score (min.; max.)		(31.8; 65.8)	(66.0; 75.7)	(75.9; 98.6)	
Age in years - average (SD)		27 (5.1)	28 (5.3)	28 (5.9)	0.01
Physical activity (min/week) - median (P25; P75)		40 (0.0; 120.0)	30 (0.0; 140.0)	60 (0.0; 140.0)	0.04
Education level - n (%)					
	≤ 3 years	9 (3.4)	7 (2.7)	11 (4.2)	0.50
	4-8 years	88 (34.1)	74 (28.1)	79 (30.3)	
	≥ 9 years	163 (62.5)	182 (69.2)	171 (65.5)	
Social class - n (%)					
	A or B	60 (23.0)	47 (17.9)	47 (18.0)	0.28
	C	174 (66.7)	178 (67.7)	174 (66.7)	
	D or E	27 (10.3)	38 (14.4)	40 (15.3)	
Self-reported race - n (%)					
	White	122 (46.8)	111 (42.2)	119 (45.6)	0.87
	Brown/Mixed	92 (35.2)	99 (37.6)	94 (36.0)	
	Other	47 (18.0)	53 (20.2)	48 (18.4)	
Marital Status - n (%)					
	Married	206 (78.9)	205 (77.9)	205 (78.5)	0.96
	Single/widow or separated	55 (21.1)	58 (22.1)	56 (21.5)	
Smoking - n (%)					
	Never smoked	202 (77.4)	210 (79.8)	212 (81.2)	0.74
	Quit during pregnancy	35 (13.4)	30 (11.4)	25 (9.6)	
	Current smoker	24 (9.2)	23 (8.8)	24 (9.2)	
Use of supplements - n (%)		120 (46.0)	161 (61.2)	214 (82.0)	< 0.001
Adequacy of BMI^b^ - n (%)					
	Low weight	7 (2.7)	13 (4.9)	11 (4.2)	0.03
	Adequate	86 (33.0)	103 (39.2)	120 (46.0)	
	Overweight	93 (35.6)	79 (30.0)	80 (30.7)	
	Obesity	75 (28.7)	68 (25.9)	50 (19.1)	
Self-reported hypertension - n (%)		26 (10.0)	18 (6.8)	32 (12.3)	0.11
Gestational diabetes^c^ - n (%)		43 (16.5)	47 (17.9)	49 (18.8)	0.79

a p-values obtained by the ANOVA test for continuous variables with normal distribution, Kruskal-Wallis for continuous variables without normal distribution, and chi-square test for categorical variables.

b Classification of current body mass index (BMI) according to gestational week (Atalah^1^, 1997).

c According to the criteria of the World Health Organization^30^ (2014).

We also observed the highest index score among the women with the highest percent of TEV derived from carbohydrates and proteins, greater intake of the vitamins C, E, and A, increased intake of calcium, folate, and iron, and lower percentage of TEV from total and saturated fat. Unexpectedly, the pregnant women categorized in the highest tertile of the score of the IQDAG had higher intake of cholesterol and lower intake of monounsaturated fats. In addition, the total energetic percentage from carbohydrates and proteins and the intake of cholesterol, vitamins, and minerals were positively correlated with IQDAG, while the percentage of TEV from total fat, saturated sat, monounsaturated fat showed negative correlation with the index, according to Spearman's correlation coefficient (Table 5). The underreporting of energy intake was verified in 47% of the participants of the study.

**Table 5:** Spearman's correlation coefficient and median (P25; P75) of the estimate of energy and nutrients of the usual diet of women according to the score of the Diet Quality Index Adapted for Pregnant Women (IQDAG). Ribeirão Preto, state of São Paulo, Brazil, 2012. (n = 785)

Variable	r	IQDAG			p^b^
		1st Tertile (n = 261)	2nd Tertile (n = 263)	3rd Tertile (n = 261)	
Energy (kcal)	0.03	2013.1 (1695.4; 2354.6)	2002.2 (1697.1; 2372.5)	2011.6 (1728.1; 2348.0)	0.778
Carbohydrates (% TEV)	0.12^a^	54.2 (50.7; 57.8)	55.0 (50.6; 59.1)	56.0 (51.8; 59.3)	0.013
Proteins (% TEV)	0.26^a^	15.7 (14.2; 17.8)	16.7 (14.6; 19.0)	17.7 (15.7; 19.6)	< 0.001
Total Fat (% TEV)	–0.16^a^	25.5 (22.0; 29.0)	24.7 (21.5; 28.1)	23.7 (20.8; 27.1)	0.010
Saturated fat (% TEV)	–0.12^a^	8.5 (7.1; 9.8)	8.3 (7.1; 9.6)	8.0 (6.9; 9.2)	0.015
Polyunsaturated fat (% TEV)	0.04	4.4 (3.5; 5.5)	4.3 (3.8; 5.1)	4.4 (3.7; 5.4)	0.690
Monounsaturated fat (% TEV)	–0.15^a^	7.5 (6.4; 8.9)	7.3 (6.2; 8.4)	7.0 (6.0; 8.2)	0.004
Trans fat (% TEV)	–0.03	1.3 (0.8; 1.9)	1.2 (0.8; 1.8)	1.2 (0.9; 1.8)	0.950
Cholesterol (mg)	0.17^a^	205.1 (164.6; 266.9)	223.5 (173.5; 282.3)	237.8 (181.6; 298.8)	< 0.001
Vitamin C (mg)	0.23^a^	157.5 (65.2; 279.5)	196.5 (89.0; 348.9)	256.3 (147.4; 401.6)	< 0.001
Vitamin E (mg)	0.13^a^	7.7 (5.7; 10.7)	8.1 (6.1; 11.4)	8.9 (6.5; 11.3)	0.012
Vitamin A (ui)	0.25^a^	10736.7 (6132.0; 16257.4)	12790.0 (7341.5; 19779.0)	15714.4 (9941.5; 21726.1)	< 0.001
Calcium (mg)	0.34^a^	419.8 (303.3; 544.9)	537.8 (412.6; 696.4)	592.9 (441.5; 759.0)	< 0.001
Folate (μg)	0.33^a^	362.0 (294.3; 421.1)	404.1 (322.1; 515.0)	452.0 (363.7; 548.6)	< 0.001
Iron (mg)	0.40^a^	10.2 (6.9; 67.0)	64.0 (8.8; 69.5)	68.3 (38.2; 72.1)	< 0.001

TEV: total energy value

a p < 0.01

b Kruskal-Wallis test.

## DISCUSSION

The IQDAG was the first quality index of the national diet to incorporate some of the guidelines of the new Dietary Guidelines for the Brazilian Population. We believe that the inclusion of the percentage of total calories from ultra-processed foods as a moderator component for this index is relevant from the public health point of view. Evidence indicates that excessive consumption of these products is positively associated with obesity and chronic diseases^21^ ^,^ ^23^. Studies also suggest that the consumption of ultra-processed foods has an impact on the cultural, social, environmental, political, and economic contexts^21^ ^,^ ^23^. In addition, diets high in these foods are nutritionally unbalanced, as they present a higher content of total fat, saturated fat, cholesterol, sodium, and added sugars and lower content of fiber, protein, and some micronutrients^29^.

Another strength was the definition of the food groups using the energy density approach (servings/1,000 kcal), differing from the other previously proposed national dietary indexes for pregnant women. The inclusion of omega-3 is also a favorable point of the IQDAG. Studies suggest that the higher intake of this micronutrient in the gestational period is inversely associated with maternal depression^6^, gestational diabetes^3^, restricted intrauterine growth^6^, and deficits in neurocognitive development^6^.

According to this index, we verified that the quality of the diet was positive in relation to the consumption of legumes and vegetables (servings/1,000 kcal), since most pregnant women reached the maximum score for these components. In Brazil, the consumption of legumes, especially bean-based preparations, is considered a marker of meal consumption (instead of the replacement for sandwiches) and thus of a healthier diet^h^. On the other hand, we observed that a low proportion of women reached the maximum score for the consumption of fresh fruits, intake of fiber, omega-3, calcium, folate, and iron, which indicates the poor quality of the diet in this aspect. This finding corroborates with a previous national study, in which pregnant women did not adopt a food intake that allowed them to reach their nutritional needs, especially regarding the intake of micronutrients^27^.

We also observed that few pregnant women reached the maximum score for the limit of consumption of ultra-processed foods, an unfavorable characteristic of the quality of the diet given the adverse effects of these foods on the health of individuals^21^ ^,^ ^23^. Similar to the data of this study, a high consumption of ultra-processed foods, such as soft drinks, crackers, and cookies, has also been found in a cross-sectional study carried out among pregnant women in the city of Botucatu, state of São Paulo, Brazil^9^.

We highlight that the diet of the mother during pregnancy can play a fundamental role in the health of the mother-child binomial. Some evidence suggests that a better quality of the diet during pregnancy is associated with a lower risk of GD^28^, as well as fetal anomalies, including neural tube defects^5^. On the other hand, specific deficiencies of micronutrients can cause low birth weight, maternal obesity, and hypertension during pregnancy^25^.

The IQDAG showed a significant correlation with the scores of all components, and we can observe the strong influence of the moderator component.

Consistent with other studies, we verified a better quality of diet among older pregnant women^4^ ^,^ ^12^ ^,^ ^18^ ^,^ ^26^, with adequate BMI^12^, who reported practicing more physical activity^18^, and who used dietary supplements^18^.

The IQDAG was sensitive to detect the quality of the diet of the pregnant women evaluated. The highest score was for the higher intake of carbohydrates and proteins (percentage of TEV), vitamins C, E, and A, and the minerals calcium, folate, and iron, as well as for the lower intake of total fats and saturated fats (percentage of TEV). Similar results have been observed in a study based on data from a cohort conducted in Singapore with 995 pregnant women using the Healthy Eating Index for Pregnant Women in Singapore (HEI-SGP)^12^ and in a study carried out in two Finnish districts that has evaluated the quality of diet of pregnant women based on the Healthy Food Intake Index (HFII)^19^. However, given the divergence of the dietary indexes for pregnant women, as well as the methods evaluating the diet used, the comparison of the results becomes limited.

Unexpectedly, pregnant women in the highest tertile of the score of the IQDAG had higher intake of cholesterol and lower intake of monounsaturated fats. A plausible explanation is that the high cholesterol foods consumed by the population of this study are also relevant sources of iron, which is a component of adequacy of the index, that is, the higher the consumption, the higher its score. As for monounsaturated fats, the pregnant women evaluated consumed few foods naturally rich in this nutrient (nuts and extra virgin olive oil). We found that the main sources of monounsaturated fat in the study population were sausage, chocolate, and margarine, which are classified as ultra-processed foods (moderator component of the index).

This study presents some limitations, the main one being the cross-sectional design. Thus, we suggest that the IQDAG should be tested for maternal and infant outcomes in different study designs. The use of the population distribution to define the cut-off points, in our case adopted for the energy percentage of ultra-processed foods, may underestimate the inadequacy of the diet reported by the study participants. However, this strategy is adopted when there is no specific recommendation for the component^11^. Another limitation inherent to dietary quality indexes is that the maximum score for the food groups is related only to the minimum number of servings consumed, with no penalty for excessive consumption. However, the fact that food groups are expressed in energy density can minimize this limitation. The underreporting of energy intake was estimated using the Goldberg formula^8^, which does not consider the physical activity of individuals and which presupposes the maintenance of body weight. Extreme values of energy intake (below or above acceptable levels) may be inherent to the gestational period, characterized both by restricted (from gastric symptoms) and high food intake (from increased appetite). In addition, we used the Estimated Average Requirement (EAR) values as a cut-off point for calcium, folate, and iron and we used the Adequate Intake (AI) for fiber and omega-3^15^. The estimation of the adequacy of nutrients without specific EAR values (fiber and omega-3) is not recommended, and the probabilistic approach is the recommended method to estimate the adequacy of iron in women of childbearing age.

However, we consider that this study proposed a relevant instrument to evaluate the quality of the diet of pregnant women, especially in the scenario of primary health care. The IQDAG was unprecedented in incorporating the recommendation on the moderation of the consumption of ultra-processed foods in a national index, thus allowing the evaluation and monitoring of the adherence of the diet of pregnant women in relation to the nutritional guidelines of the current Brazilian dietary guidelines. Unlike previous national indexes for pregnant women, we also considered the energy density approach in the definition of food groups. We found the highest index score among older and eutrophic women who reported a healthy lifestyle. In addition, our findings reinforce that strategies are needed to promote the consumption of fresh fruits, foods high in fiber, omega-3, calcium, folate, iron, and fresh or minimally processed foods among pregnant women.

## Footnotes

a Malta MB. Avaliação da alimentação de gestantes mediante aplicação do índice de Qualidade da Dieta adaptado [dissertation]. Botucatu: Faculdade de Medicina de Botucatu, Universidade Estadual Paulista “Júlio de Mesquita Filho”; 2010.

b Ministério da Saúde (BR), Secretaria de Atenção à Saúde, Departamento de Atenção Básica. Guia alimentar para população brasileira. 2. ed. Brasília (DF); 2014 [cited 2018 Feb 7]. Available from: http://bvsms.saude.gov.br/bvs/publicacoes/guia_alimentar_populacao_brasileira_2ed.pdf

c Associação Brasileira de Empresa de Pesquisa. Dados com base no levantamento socioeconômico 2012 - IBOPE. São Paulo; ABEP; 2012 [cited 2018 Feb 7]. Available from: http://www.abep.org

d Universidade Estadual de Campinas, Núcleo de Estudos e Pesquisas em Alimentação. Tabela brasileira de composição de alimentos - TACO. 2.ed. Campinas: NEPA; 2006 [cited 2018 Feb 7]. Available from: http://www.crn1.org.br/wp-content/uploads/2015/04/taco.pdf?x53725

e USDA National Nutrient Database for Standard Reference. Beltsville: U.S. Departament of Agriculture, Agricultural Research Service Research; 2001. Available from: https://www.ars.usda.gov/northeast-area/beltsville-md/beltsville-human-nutrition-research-center/nutrient-data-laboratory/docs/sr14-home-page

f NutWin - Programa de Apoio à Nutrição. Versão 1.5. São Paulo: Escola Paulista de Medicina; 2002.

g Ministério da Saúde (BR), Secretaria de Atenção à Saúde, Departamento de Atenção Básica. Atenção ao pré-natal de baixo risco. Brasília (DF); 2012 [cited 2018 Feb 7]. (Série A. Normas e Manuais Técnicos) (Cadernos de Atenção Básica, 32). Available from: http://bvsms.saude.gov.br/bvs/publicacoes/cadernos_atencao_basica_32_prenatal.pdf
